# Patrones de hipercementosis y su relación con posibles factores etiológicos locales en radiografías de individuos de una población mexicana

**DOI:** 10.21142/2523-2754-1103-2023-163

**Published:** 2023-09-26

**Authors:** María Alejandra Bernal Ruiz, Gustavo Adolfo Fiori Chíncaro

**Affiliations:** 1 Imagen y Diagnóstico Dental (IMADENT). Aguascalientes, México. alejandrabernalrz@hotmail.com Imagen y Diagnóstico Dental (IMADENT) Aguascalientes México alejandrabernalrz@hotmail.com; 2 División de Radiología Bucal y Maxilofacial, Universidad Científica del Sur. Lima, Perú Universidad Científica del Sur División de Radiología Bucal y Maxilofacial Universidad Científica del Sur Lima Peru; 3 Instituto Latinoamericano de Altos Estudios en Estomatología (ILAE). Lima, Perú. gfiori@ilaeperu.com Instituto Latinoamericano de Altos Estudios en Estomatología (ILAE) Lima Perú gfiori@ilaeperu.com

**Keywords:** hipercementosis, radiografía dental, prevalencia (DeCS), hypercementosis, radiography, dental, prevalence (DeCS)

## Abstract

**Objetivo:**

La hipercementosis (HPC) es una patología asintomática que, según la literatura existente, presenta una prevalencia baja, por lo que poco se ha investigado sobre ella; entre estos estudios, son pocos los elaborados para grupos étnicos. El objetivo de esta investigación fue determinar la prevalencia y los patrones radiográficos de dicha condición, así como el análisis de la relación de la patología con algunos de los que son considerados posibles factores locales desencadenantes (FDL) en individuos mexicanos.

**Metodología:**

Se analizaron 1193 ortopantomografías (OPG), seleccionadas de manera aleatoria de pacientes de ambos sexos, con un rango edad cronológica entre los 18 y 90 años, identificando la prevalencia de HPC, así como su relación entre grupos etarios, sus patrones morfológicos (focal, difuso y en forma de manga), su distribución por región anatómica y órgano dental (ODs), y la asociación de su presencia con posibles factores desencadenantes locales.

**Resultados:**

Se encontraron 348 OD con HPC en un total de 194 pacientes (16,30%), sin diferencias relevantes entre género (p > 0,05). Se registró un aumento significativo respecto de la presencia de HPC en relación con el incremento de la edad de los pacientes (p = 0,001), encontrándola presente en el 10% del grupo etario < 40 años, en un 20;30 % en el grupo de 40 a 60 años y en un 30.20% en > 60. Se encontró con mayor frecuencia de forma difusa (75,28 %), seguida por el patrón focal (19,54%) y fue menos común la morfología en forma de manga (5,17%). La mandíbula presentó la mayor cantidad de OD con presencia de HPC, 136 (39,08%), y el lado izquierdo fue el más afectado, con 86 OD. El grupo dentario con mayor afectación fue el de molares y premolares.

**Conclusiones:**

La prevalencia de hipercementosis fue del 16,30% en los individuos mexicanos evaluados. Su presencia aumenta al incrementar la edad de los pacientes. Su localización principal es la región mandibular con predilección de premolares y molares. A pesar de que el origen idiopático es el más frecuente, se observó que la impactación dental es un posible factor local desencadenante.

## Abstract (en)

**Objective:**

Hypercementosis (HPC) is an asymptomatic pathology that, according to the existing literature, has a low prevalence, there is a lack of information and research on it, within these studies, few are made by ethnic groups. To determine the prevalence and radiographic patterns of this condition, as well as the analysis of the relationship of the pathology with some of what are considered possible local triggering factors (FDL) in Mexican individuals.

**Methodology:**

1193 orthopantomographies (OPG) were analyzed, randomly selected from patients of both sexes, with a chronological age range between 18 and 90 years, identifying the prevalence of HPC, as well as its relationship between age groups, its morphological patterns (focal, diffuse and sleeve-shaped), its distribution by anatomical region and dental organs (ODs) and the association of its presence with possible local triggering factors.

**Results:**

348 DO with HPC were found in a total of 194 patients (16.30%), with no relevant differences between genders (P> 0.05). There was a significant increase with respect to the presence of HPC in relation to the increase in the age of the patients (P= 0.001), finding it present in 10% of the age group <40 years, in 20.30% in the group of 40 to 60 years and > 60 in 30.20%. It was found more frequently in a diffuse form (75.28%), followed by the focal pattern (19.54%) and finding the sleeve-shaped morphology less common (5.17%). The mandible presented the greatest number of ODs with the presence of HPC, 136 (39.08%), with the left side being the most affected with 86 OD. The dental group with the greatest involvement was that of molars and premolars.

**Conclusions:**

The prevalence of hypercementosis was 16.30% in the Mexican individuals evaluated. Its presence increases as the age of the patients advances. Its main location is the mandibular region with a predilection for premolars and molars. Even though the idiopathic origin is the most frequent, it was observed that dental impaction is a possible local triggering factor.

## INTRODUCCIÓN

El cemento es un tejido muy delgado, mineralizado y no vascularizado que cubre la totalidad de la superficie radicular ^1^^-^^4^. Las células responsables de la cementogénesis (cementoblastos, cementocitos y fibroblastos) provienen de células ectomesenquimales ^5^^-^^8^.

Por mucho tiempo se ha considerado que la función primordial y única del cemento es proveer un medio de anclaje y soporte de las fibras colágenas del ligamento periodontal al diente ^4^^,^^8^; pero, con el avance de las investigaciones y estudios, se ha comprobado que es un componente adaptativo del periodonto que puede responder a cambios funcionales en la oclusión e, incluso, es denominado como el tejido de reparación (mediante aposición de capas) en fracturas o reabsorciones radiculares ^8^^,^^10^.

La hipercementosis (HPC) se define como una condición no neoplásica de aposición excesiva de cemento radicular secundario, que puede encontrarse de forma aislada en un órgano dental (OD), considerándola como HPC localizada, o presentarse en múltiples órganos dentales, considerándola como HPC generalizada ^6^^,^^11^^-^^14^.

**Figura 1:**
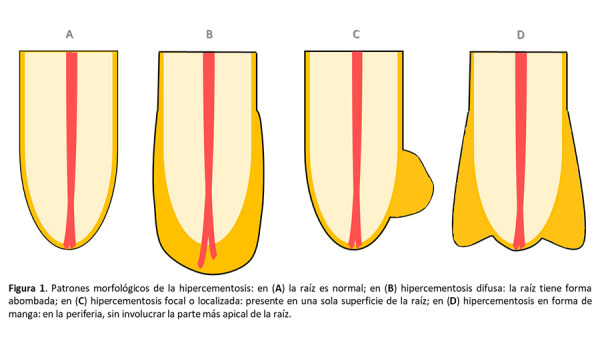
Patrones morfólogicos de la hipercementosis: en (A) la raíz es normal; en (B) hipercementosis difusa: la raíz tiene forma abombada; en (C) hipercementosis focal o localizada: presente en una sola superficie de la raíz; en (D) hipercementosis en forma de manga: en la periferia, sin involucrar la parte más apical de la raíz.

Respecto de los patrones morfológicos, se encuentran tres tipos: difusa (la raíz tiene una forma abombada), focal o localizada (en una sola superficie de la raíz) y en forma de manga (en la periferia, sin involucrar la parte más apical de la raíz, como resultado de una lesión periapical) ^14^^-^^17^ (Fig. 1).

**Figura 2:**
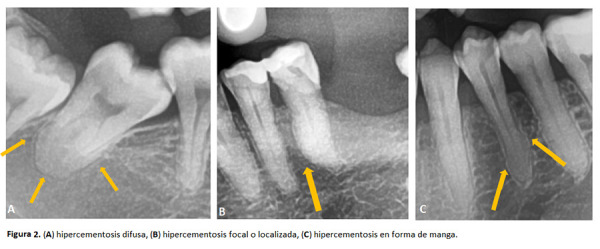
(A) hipercementosis difusa, (B) hipercementosis focal o localizada, (C) hipercementosis en forma de manga.

**Figura 3:**
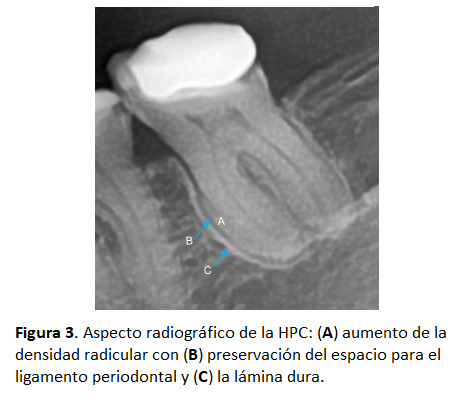
Aspecto radiográfico de la HPC: **(A)** aumento de la densidad radicular con **(B)** preservación del espacio para el ligamento periodontal y **(C)** la lámina dura.

Su prevalencia fluctúa del 1,3% al 8% de la población y tiene mayor presencia en adultos jóvenes y adultos mayores, con mayor incidencia en zona de molares y premolares ^8^^,^^18^^,^^19^. Aunque es considerada una entidad idiopática, se asocia a factores locales como extrusión dental por falta de antagonista, trauma oclusal e inflamación periapical, y a factores sistémicos como: ateroesclerosis, artritis, enfermedades tiroideas, gigantismo y enfermedad de Paget ^3^^,^^6^^-^^9^^,^^11^^,^^13^^,^^16^.

Generalmente, se presenta de forma asintomática como un hallazgo radiográfico. Se observa como un engrosamiento del contorno radicular de forma parcial o total, en donde la radiolucidez del espacio del ligamento periodontal y la radiopacidad de la lámina dura acompañan siempre el borde externo de la HPC, considerándose ambos elementos anatómicos como preservados ^8^^,^^9^^,^^11^^,^^20^.

El principal diagnóstico diferencial de la HPC (en presencia excesiva) es el cementoblastoma, lo que se debe a la similitud de las características radiográficas (preservación del espacio del ligamento periodontal y lámina dura), aunque es relacionada también con alteraciones radiopacas de asociación estrecha con las raíces dentales, tales como osteoesclerosis idiopática, osteítis condensante, displasia ósea, fibroma osificante, osteoma central y osteoma osteoide ^21^^,^^22^.

Los OD con presencia de HPC no requieren tratamiento, pero su detección y evaluación es relevante al momento de realizar tratamientos en especialidades como endodoncia, al variar la longitud y ubicación del formen apical ^7^^,^^23^^,^^24^; en ortodoncia, al ocasionar dificultad en el movimiento del diente por anquilosis parcial ^25^, y en exodoncia, al condicionar la extracción simple de OD que presenten dicha alteración y terminar en un procedimiento quirúrgico. En ese sentido, el propósito de este estudio fue determinar la prevalencia y patrones radiográficos de la HPC, así como la búsqueda de relación entre posibles factores locales que puedan desencadenar su presencia en individuos de una población mexicana.

## MATERIALES Y MÉTODOS

### Diseño del estudio y cálculo de la muestra

Se realizó una investigación de tipo observacional, transversal y retrospectiva. La muestra estuvo constituida por 1,193 OPG de pacientes mayores de edad (597 de sexo femenino y 596 de sexo masculino), adquiridas en el periodo de abril de 2017 a abril de 2022, en un centro radiológico privado de la ciudad de Aguascalientes, México, las cuales fueron seleccionadas de manera aleatoria simple de la base de datos digital del centro. 

El cálculo de muestra fue obtenido mediante el *softwareT* Open Epi, versión 3, (https://www.openepi.com/SampleSize/SSPropor.htm). Se estableció un intervalo de confianza del 99,99% en una población de 32,492, con una frecuencia anticipada del 8% de presencia de hipercementosis en la población y un margen de error del +/-3%. 

### Adquisición y lectura de las imágenes

Las imágenes radiográficas fueron adquiridas con el equipo panorámico digital directo de Marca Vatech INC (Corea del Sur), modelo PaX-i3D Green PHT-60CFO, utilizando valores de adquisición de 14 mA y 75 Kvp. De acuerdo con el protocolo recomendado por el fabricante, fueron procesadas con un software especializado (EzDent-i, ver.3.0.10.0 Client, Ewoosoft Co., Ltd.) y visualizadas en un monitor curvo HD de 27” marca Samsung modelo CR50, y una PC (laptop) de marca MSI, modelo GF63 Thin 9SCSR, procesador Intel Core i5 9.a generación, con tarjeta gráfica NVIDIA GeForce GTX 1650 Ti. 

Se garantizó el anonimato de los sujetos de acuerdo con los lineamientos de la Declaración de Helsinki. Asimismo, se contó con la aprobación del Comité Institucional de Ética e Investigación de la Universidad Científica del Sur (POS-117-2022-00347). Para esta investigación, ningún paciente fue expuesto a radiación ionizante, ya que el examen se indicó por causas clínicas.

### Medición de las variables y recolección de datos

Los datos demográficos evaluados fueron el género y la edad de los participantes. Se consideró como HPC cuando se observó aposición excesiva de cemento en el contorno radicular de los OD, en donde la radiolucidez del espacio del ligamento periodontal y la radiopacidad de la lámina dura acompañan siempre el borde externo de la HPC, considerándose ambos elementos anatómicos como preservados. 

Se registró su localización de acuerdo con el número de OD, según la clasificación de la FDI, considerando en números enteros la cantidad de dientes afectados.

Se consideró como morfología de la HPC (esquema) al patrón de forma en la aposición de cemento en la zona radicular: a) difusa (la raíz tiene una forma abombada), b) focal o localizada (en una sola superficie de la raíz), y c) en forma de manga (en la periferia, sin involucrar la parte más apical de la raíz).

Los posibles factores desencadenantes locales fueron determinados como la exposición del OD a alguna alteración local que pueda ser predisponente a que este desarrolle HPC. Se consideró los siguientes: a) Tratamiento endodóntico: tratamiento de conductos orientado a preservar un órgano dental que tiene la pulpa inflamada o infectada de forma irreversible; b) Facetamiento en la estructura coronal del órgano dental, desgaste presente en las superficies incisales y oclusales; c) Impactación dental: órgano dental cuyo ápice se encuentra totalmente cerrado y que no se encuentra erupcionado (total o parcialmente), debido a la existencia de una barrera física; d) Extrusión dental: erupción continua de un órgano dental que no presenta antagonista, y e) Inflamación apical: imagen radiolúcida en relación con la zona apical del órgano dental.

### Análisis de datos

Se realizó un plan de capacitación y evaluación de resultados a través de una prueba piloto, en la que participó el investigador y como *gold standard* un especialista en radiología bucal y maxilofacial con 10 años de experiencia. Se realizaron pruebas de concordancia inter e intra examinadores, y se consideró apto al tener una evaluación a través del estadístico Kappa mayor a 0.8., utilizando el 10% de la muestra. Mediante el uso del programa SPPS para Windows versión 19.0, se aplicaron los test exactos de Fisher y la prueba de chi cuadrado para evaluar la existencia de alguna asociación. El valor de p fue significativo a p < 0,05.

## RESULTADOS

Se realizó el análisis de 1193 radiografías panorámicas, las cuales 597 pertenecen al sexo femenino y 596 al masculino. La media/desviación estándar (DS) de la edad en el género femenino fue de 41,6 ± 15,933, mientras que en el masculino se registró en 39,82 ± 16,327 años (p = 0,057). Se encontraron 348 OD con HPC en un total de 194 pacientes (16,30%), de los cuales 103 (17,30 %) corresponden al género femenino y 91(15,30%), al género masculino (p = 0,198) (Tabla 1).

**Tabla 1:** Asociación entre la prevalencia de hipercementosis y el grupo etario en la muestra evaluada

Género	HPC ausente	HPC presente	Total
	n (%)	n (%)	n (%)
Femenino	494 (82,70)	103 (17,30)	597 (100)
Masculino	505 (84,70)	91 (15,30)	596 (100)
Total	999 (83,70)	194 (16,30)	1193 (100)

Valor de p = 0,198 Prueba exacta de Fisher

Se registró un aumento significativo con respecto a la presencia de HPC en relación con el incremento de la edad de los pacientes (p = 0,001), y se halló presente en el 10% del grupo etario de < 40 años, el 20,30 % en el grupo de 40 a 60 años, y el 30,20% en el grupo > 60 (Tabla 2).

**Tabla 2:** Asociación entre la prevalencia de HPC y el grupo etario

Edad	HPC ausente	HPC presente	Total
	n (%)	n (%)	n (%)
< 40	561 (90,00)	62 (10,00)	623 (100)
40 a 60	325 (79,70)	83 (20,30)	408 (100)
> 60	113 (69,80)	49 (30,20)	162 (100)
Total	999 (83,70)	194 (16,30)	1194 (100)

Valor de p = 0,001. Prueba de chi cuadrado

Respecto de los patrones morfológicos, se encontró que la forma difusa es la más frecuente (75,28%), seguida por el patrón focal (19,54%) y fue menos común la morfología en forma de manga (5,17%) (Tabla 3).

**Tabla 3:** Patrones morfológicos de HPC

Patrón	Frecuencia	Porcentaje
Difusa	262	75,28
Focal	68	19,54
Manga	18	5,17
Total	348	100

En relación con los posibles factores desencadenantes locales (FDL) asociados con HPC, encontramos que la ausencia de HPC (factor idiopático) resultó la más común (36,4 %), seguida por la impactación (23,3%), el facetamiento (16,7 %), la extrusión (11%), la presencia de tratamiento endodóntico en el OD (8,2%) y la inflamación apical (4,4%). Se encontraron 17 OD (4,8%), con presencia de más de un FDL (Tabla 4).

**Tabla 4:** Frecuencia de posibles FDL en OD con HPC

FDL	Frecuencia	Porcentaje
Ausente	133	36,4
Endodoncia	30	8,2
Facetamiento	61	16,7
Impactación	85	23,3
Extrusión	40	11
Inflamación apical	16	4,4
Total	365	100

Se observó una media de 1,79 ± 1,234 de OD con presencia de HPC en los pacientes, con hasta 9 dientes afectados en un mismo paciente. El 55,67% (108 pacientes) presentaron HPC localizada, mientras que en el 44,32% (86 pacientes) se observaron dos o más OD afectados.

La mandíbula presentó la mayor cantidad de OD con presencia de HPC, con 136 (39,08%), y fue el lado izquierdo el más afectado, con 86 OD. El grupo dentario con mayor afectación fue el de molares y premolares (Tabla 5).

**Tabla 5:** Distribución de HPC por región anatómica y grupo dentario

Región	Grupo dentario	Frecuencia	Porcentaje
Maxilar	Incisivos	1	0,28
	Caninos	17	4,88
	Premolares	23	6,6
	Molares	47	13,5
	Total	88	25,28
Mandíbula	Incisivos	4	1,14
	Caninos	33	9,48
	Premolares	60	17,24
	Molares	163	46,83
	Total	260	74,71
Total		348	100

## DISCUSIÓN

En la literatura existen pocos estudios que evalúen la prevalencia de la HPC en grupos poblacionales y estos han mostrado resultados fluctuantes: el 1,3% de la población alemana ^8^, el 9,8% en un grupo evaluado en población femenina saudí ^15^ y el 10,8 % en una población turca ^12^. El presente estudio muestra un aumento significativo (16,30%) y la diferencia entre los resultados puede estar relacionada con las diferencias poblacionales y los métodos de evaluación de cada uno de los estudios. Al respecto, Bürklein *et al*. ^8^ realizaron el análisis en radiografías periapicales, mientras que los estudios de Elsayed y Defne ^12^^,^^15^ evaluaron la prevalencia de HPC en radiografías panorámicas, y encontraron parámetros más fiables para la comparación de resultados.

La HPC es definida de manera uniforme en la literatura como una afección asintomática cuyo diagnóstico se establece radiográficamente al observar un engrosamiento del contorno radicular de forma parcial o total, en donde la radiolucidez del espacio del ligamento periodontal y la radiopacidad de la lámina dura acompañan siempre el borde externo de la HPC, considerándose ambos elementos anatómicos como preservados, y esta característica es la clave para su diagnóstico diferencial ^8^^,^^9^^,^^11^^,^^20^, criterio que fue utilizado dentro del estudio para su evaluación.

En el presente estudio, al igual que en el estudio llevado a cabo en poblaciones turcas ^7^^,^^12^, no hubo datos significativos con respecto a la diferencia de género entre los evaluados (p = 0,198). Dieter y Knut ^26^ definieron al cemento como un tejido dinámico que aumenta su grosor con la edad y en función a las demandas del periodonto y los cambios en la estructura y función de los órganos dentales. En cuanto a lo anterior, se registró un aumento significativo respecto de la presencia de HPC en relación con el incremento de la edad de los pacientes (p = 0,001), y se encontró presente en mayor porcentaje en el grupo etario mayor a 60 años (30.20%), lo que coincide con los resultados de Defne *et al*. ^12^ (p = 0,022).

Consolaro *et al*. ^17^ clasificaron morfológicamente a la HPC entre grupos: difusa, con aspecto abombado alrededor de todo el contorno radicular; focal, localizada en una de las superficies de la raíz; en forma de manga, en ambas superficies radiculares sin involucrar la zona apical. El presente estudio muestra a la entidad morfológica difusa como la más prevalente (75,28%), lo cual fue reportado de la misma forma por Elsayed *et al*. ^12^ y Pinheiro BC *et al*. ^27^.

Aunque la HPC es considerada una entidad idiopática, se ha buscado establecer relación con factores desencadenantes tanto sistémicos como locales. En el presente estudio no contamos con información clínica de los pacientes, por lo que solamente fueron evaluados factores locales observados en las OPG. A pesar de que el factor idiopático fue el más común (36,4%), es importante destacar que se observó un número importante de OD impactados con presencia de HPC (23,3%), lo que coincide con la teoría de Massé *et al*. ^28^, la cual señala que la presencia de HPC no solo se limita a OD ya erupcionados, de modo que establece una posible asociación con fuerzas de erupción presentes en OD sin erupcionar y con la presencia de alguna barrera física que dificulte su erupción. En el estudio, se observaron también OD con facetamiento en el 16,7%; extrusión, con un 11%; presencia de tratamiento endodóntico en el OD, con un 8,2%, e inflamación apical, con el 4,4%.

En coincidencia con los estudios poblacionales que evaluaron la HPC ^8^^,^^12^^,^^15^, se encontró que la mandíbula presentó la mayor cantidad de OD con HPC, 136 (39,08%), y que el grupo dentario con mayor afectación fue el de molares y premolares, pero difiere de Elsayed *et al*. ^12^, al observar en nuestro estudio que el lado izquierdo fue el más afectado, con 86 OD. 

## CONCLUSIÓN

A pesar de existir información vasta en la literatura acerca de la HPC, son pocos los estudios poblacionales que permiten realizar un estudio a profundidad sobre esta, por lo que se encontró dificultad para la comparación de resultados y el establecimiento de criterios uniformes que permitan una mejor evaluación y correlación clínica.

El presente estudio pretende establecer un criterio más completo de la HPC, en el que, además de estudiar la prevalencia en individuos de una población mexicana, puedan evaluarse los patrones radiográficos para su adecuado diagnóstico y clasificación, tales como sus patrones morfológicos, de localización, así como posibles factores locales que puedan inducir su aparición.

Es necesario continuar con estudios de esta índole en diferentes poblaciones para, mediante su análisis y correlación, establecer clasificaciones uniformes que permitan una evaluación completa y así identificar de manera certera los factores tanto locales como sistémicos que desencadenan la HPC.

## Fuente de financiamiento:

Autofinanciado.
